# 4,4′-Dibromo-7,7′-dimeth­oxy-1,1′-spiro­biindane

**DOI:** 10.1107/S1600536810046519

**Published:** 2010-11-17

**Authors:** Min Yao, Yanfeng Ding, Zi-jia Wang, Yuheng Deng

**Affiliations:** a Department of Chemistry, Capital Normal University, Beijing 100048, People’s Republic of China

## Abstract

In the title compound, C_19_H_18_Br_2_O_2_, the dihedral angle between the two benzene rings of the spiro­biindane molecule is 70.44 (8)°. In the crystal, mol­ecules are inter­connected along the *c* axis by C—H⋯O hydrogen bonds and π–π stacking [centroid–centroid distance = 3.893 (2) Å] inter­actions, forming an infinite chain structure. The chains are further inter­connected through another set of C—H⋯O hydrogen bonds, forming layers approximately parallel to the *bc* plane.

## Related literature

For studies on spiranes, see: Srivastava *et al.* (1992 ▶); Chan *et al.* (1997 ▶); Ding *et al.* (2009 ▶). For 1,1′-spiro­biindane and its analogs, see: Brewster & Prudence (1973 ▶); Birman *et al.* (1999 ▶). 
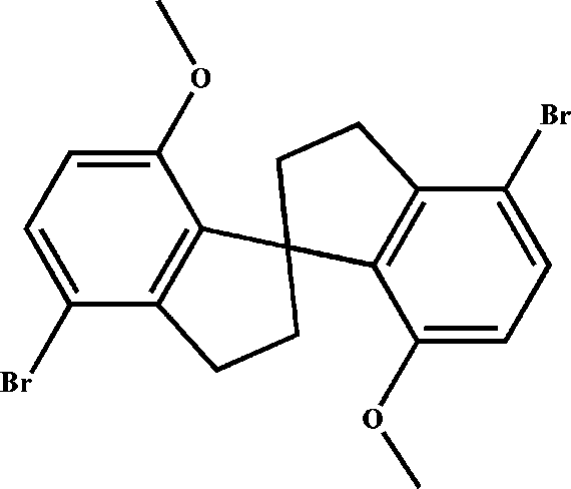


## Experimental

### 

#### Crystal data

C_19_H_18_Br_2_O_2_
*M* *_r_* = 438.15
Triclinic, 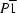

*a* = 8.3487 (3) Å
*b* = 10.4831 (3) Å
*c* = 11.6293 (4) Å
α = 112.047 (2)°
β = 105.559 (2)°
γ = 94.280 (2)°
*V* = 891.11 (5) Å^3^
*Z* = 2
Mo *K*α radiation
μ = 4.56 mm^−1^
*T* = 296 K
0.40 × 0.16 × 0.10 mm

#### Data collection

Bruker APEXII CCD area-detector diffractometer
Absorption correction: multi-scan (*SADABS*; Bruker, 2007 ▶) *T*_min_ = 0.263, *T*_max_ = 0.659
9917 measured reflections
3090 independent reflections
2470 reflections with *I* > 2σ(*I*)
*R*_int_ = 0.016

#### Refinement

*R*[*F* ^2^ > 2σ(*F* ^2^)] = 0.030
*wR*(*F* ^2^) = 0.081
*S* = 1.05
3090 reflections
208 parameters
H-atom parameters constrained
Δρ_max_ = 0.51 e Å^−3^
Δρ_min_ = −0.41 e Å^−3^

### 

Data collection: *APEX2* (Bruker, 2007 ▶); cell refinement: *APEX2* and *SAINT* (Bruker, 2007 ▶); data reduction: *SAINT*; program(s) used to solve structure: *SHELXS97* (Sheldrick, 2008 ▶); program(s) used to refine structure: *SHELXL97* (Sheldrick, 2008 ▶); molecular graphics: *SHELXTL* (Sheldrick, 2008 ▶); software used to prepare material for publication: *SHELXTL* and *PLATON* (Spek, 2009 ▶).

## Supplementary Material

Crystal structure: contains datablocks I, global. DOI: 10.1107/S1600536810046519/zq2071sup1.cif

Structure factors: contains datablocks I. DOI: 10.1107/S1600536810046519/zq2071Isup2.hkl

Additional supplementary materials:  crystallographic information; 3D view; checkCIF report

## Figures and Tables

**Table 1:** Hydrogen-bond geometry (Å, °)

*D*—H⋯*A*	*D*—H	H⋯*A*	*D*⋯*A*	*D*—H⋯*A*
C18^i^—H18*A*^i^⋯O1	0.96	2.56	3.416 (6)	149
C19^ii^—H19*A*^ii^⋯O2	0.96	2.52	3.365 (2)	147

Symmetry codes: (i) 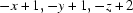; (ii) 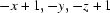.

**Fig. 1.**
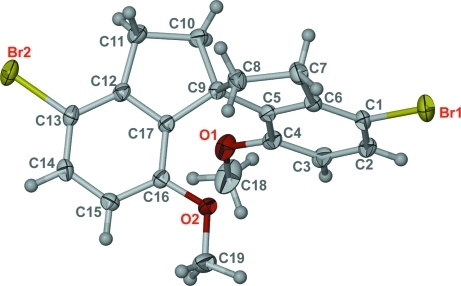
The atom-numbering scheme of the title compound. Displacement ellipsoids are shown at 30% probability level. All hydrogen atoms are omitted for clarity.

**Fig. 2.**
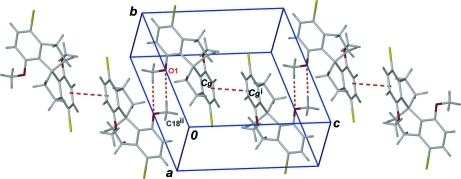
Crystal packing showing the C-H···O and π–π interactions along the c direction forming infinite chains (symmetry codes: (i) -x+1, -y+1, -z+1; (ii) -x+1, -y+1, -z+2).

**Fig. 3.**
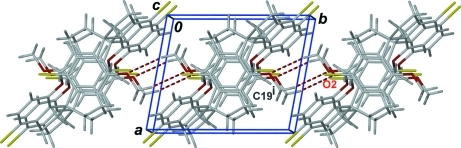
Crystal packing showing the C-H···O hydrogen bonds bridging the infinite chains (symmetry code: (i) -x+1, -y, -z+1).

## supplementary crystallographic information

### Comment

Spiranes are typical molecules with axial chirality. Spirane derivatives have been mainly employed in ligand design and asymmetric synthesis (Srivastava *et al.*,1992; Chan *et al.*,1997; Ding *et al.*, 2009). Among them, 1,1'-spirobiindane and its analogs have also attracted much attention for their featuring *C_2_*-symmetric chiral property (Birman *et al.* , 1999; Brewster *et al.*, 1973). In the present context, we report the structure of a known compound 4,4'-dibromo-7,7'-dimethoxy-1,1'-spirobiindane, a derivative of 1,1'-spirobiindane.

In the crystal structure of the title compound, C_19_H_18_Br_2_O_2_, the dihedral angle between the two phenyl rings of the spirobiindane moieties is 70.44 (8)° (Fig. 1). The molecules are arranged along the *c* axis and linked through C-H···O hydrogen bonds (C18^ii^-H18A^ii^(methyl)···O1 with D···A = 3.416 (2) Å, H···A = 2.56, and D-H···A 148.5°) and π(benzene)···π(benzene) stacking interactions (*C_g_*···*C_g_*^i^ = 3.893 (2) Å) forming an infinite chain structure [Fig. 2, symmetry codes: (i) -x+1, -y+1, -z+1 (ii) -x+1, -y+1, -z+2]. The formed chains are further interconnected by an other set of C-H···O hydrogen bonds [C19^i^-H19A^i^···O2 with D···A = 3.365 (2) Å, H···A = 2.52, and D-H···A 146.9°: (i) -x+1, -y, -z+1] to form layers approximately parallel to the *bc* plane, as shown in Fig. 3.

### Experimental

The title compound was prepared following the literature procedure (Birman *et al.* , 1999). The 1,5-bis-(2-bromo-5-methoxyphenyl)-3-pentanone was stirred with polyphosphoric acid at 105°C to obtain the title compound as the main product. The crude compound was purified by column chromatography on silica gel (hexane/EtOAc = 9:1 v.v), yield 65%. The orange crystals of the title compound having an average 0.40 × 0.16 × 0.10 mm dimension were obtained by slow evaporation from its solution of hexane.

### Refinement

The H atoms were placed in idealized positions and allowed to ride on the relevant carbon atoms, with C-H = 0.93 and 0.97 Å for aryl and methylene H atoms, respectively, and U*_iso_*(H) = 1.2U*_eq_*(C).

### Crystal data

**Table:**

C_19_H_18_Br_2_O_2_	*Z* = 2
*M**_r_* = 438.15	*F*(000) = 436
Triclinic, *P*1	*D*_x_ = 1.633 Mg m^−^^3^
*a* = 8.3487 (3) Å	Mo *K*α radiation, λ = 0.71073 Å
*b* = 10.4831 (3) Å	Cell parameters from 9917 reflections
*c* = 11.6293 (4) Å	θ = 2.2–25.0°
α = 112.047 (2)°	µ = 4.56 mm^−^^1^
β = 105.559 (2)°	*T* = 296 K
γ = 94.280 (2)°	Block, orange
*V* = 891.11 (5) Å^3^	0.40 × 0.16 × 0.10 mm

### Data collection

**Table:**

Bruker APEXII CCD area-detector diffractometer	3090 independent reflections
Radiation source: fine-focus sealed tube	2470 reflections with *I* > 2σ(*I*)
graphite	*R*_int_ = 0.016
ω scans	θ_max_ = 25.0°, θ_min_ = 2.2°
Absorption correction: multi-scan (*SADABS*; Bruker, 2007)	*h* = −9→9
*T*_min_ = 0.263, *T*_max_ = 0.659	*k* = −12→12
9917 measured reflections	*l* = −12→13

### Refinement

**Table:**

Refinement on *F*^2^	Primary atom site location: structure-invariant direct methods
Least-squares matrix: full	Secondary atom site location: difference Fourier map
*R*[*F*^2^ > 2σ(*F*^2^)] = 0.030	Hydrogen site location: inferred from neighbouring sites
*wR*(*F*^2^) = 0.081	H-atom parameters constrained
*S* = 1.05	*w* = 1/[σ^2^(*F*_o_^2^) + (0.0381*P*)^2^ + 0.4917*P*] where *P* = (*F*_o_^2^ + 2*F*_c_^2^)/3
3090 reflections	(Δ/σ)_max_ = 0.001
208 parameters	Δρ_max_ = 0.51 e Å^−^^3^
0 restraints	Δρ_min_ = −0.41 e Å^−^^3^

### Special details

**Table:**

Geometry. All esds (except the esd in the dihedral angle between two l.s. planes) are estimated using the full covariance matrix. The cell esds are taken into account individually in the estimation of esds in distances, angles and torsion angles; correlations between esds in cell parameters are only used when they are defined by crystal symmetry. An approximate (isotropic) treatment of cell esds is used for estimating esds involving l.s. planes.
Refinement. Refinement of F^2^ against ALL reflections. The weighted R-factor wR and goodness of fit S are based on F^2^, conventional R-factors R are based on F, with F set to zero for negative F^2^. The threshold expression of F^2^ > 2sigma(F^2^) is used only for calculating R-factors(gt) etc. and is not relevant to the choice of reflections for refinement. R-factors based on F^2^ are statistically about twice as large as those based on F, and R- factors based on ALL data will be even larger.

### Fractional atomic coordinates and isotropic or equivalent isotropic displacement parameters (Å^2^)

**Table:**

	*x*	*y*	*z*	*U*_iso_*/*U*_eq_	
Br1	1.10416 (5)	−0.01254 (4)	0.77083 (5)	0.08981 (17)	
Br2	0.47345 (5)	0.80256 (3)	0.72566 (4)	0.07264 (15)	
O1	0.5993 (3)	0.3495 (2)	0.9166 (2)	0.0653 (6)	
O2	0.4765 (2)	0.18707 (19)	0.5868 (2)	0.0529 (5)	
C1	0.9460 (4)	0.1024 (3)	0.8190 (3)	0.0584 (8)	
C2	0.8577 (4)	0.0773 (3)	0.8944 (3)	0.0627 (9)	
H2A	0.8767	0.0061	0.9227	0.075*	
C3	0.7404 (4)	0.1577 (3)	0.9285 (3)	0.0578 (8)	
H3A	0.6811	0.1406	0.9802	0.069*	
C4	0.7105 (4)	0.2636 (3)	0.8863 (3)	0.0502 (7)	
C5	0.7982 (3)	0.2868 (3)	0.8074 (3)	0.0442 (6)	
C6	0.9173 (3)	0.2067 (3)	0.7741 (3)	0.0503 (7)	
C7	0.9965 (4)	0.2507 (4)	0.6906 (4)	0.0648 (9)	
H7A	1.0008	0.1700	0.6160	0.078*	
H7B	1.1103	0.3053	0.7403	0.078*	
C8	0.8773 (4)	0.3406 (4)	0.6466 (3)	0.0584 (8)	
H8A	0.7945	0.2843	0.5614	0.070*	
H8B	0.9414	0.4179	0.6410	0.070*	
C9	0.7877 (3)	0.3968 (3)	0.7517 (3)	0.0453 (6)	
C10	0.8785 (4)	0.5453 (3)	0.8581 (3)	0.0596 (8)	
H10A	0.8676	0.5560	0.9420	0.072*	
H10B	0.9981	0.5610	0.8671	0.072*	
C11	0.7909 (4)	0.6495 (3)	0.8117 (3)	0.0605 (8)	
H11A	0.7849	0.7308	0.8851	0.073*	
H11B	0.8498	0.6804	0.7625	0.073*	
C12	0.6173 (4)	0.5641 (3)	0.7261 (3)	0.0446 (6)	
C13	0.4707 (4)	0.6077 (3)	0.6789 (3)	0.0477 (7)	
C14	0.3237 (4)	0.5120 (3)	0.6013 (3)	0.0533 (7)	
H14A	0.2257	0.5423	0.5707	0.064*	
C15	0.3202 (4)	0.3699 (3)	0.5681 (3)	0.0498 (7)	
H15A	0.2200	0.3052	0.5150	0.060*	
C16	0.4663 (3)	0.3244 (3)	0.6142 (3)	0.0420 (6)	
C17	0.6141 (3)	0.4225 (3)	0.6941 (2)	0.0394 (6)	
C18	0.5014 (7)	0.3249 (5)	0.9914 (6)	0.1131 (17)	
H18A	0.4297	0.3928	1.0065	0.170*	
H18B	0.5755	0.3330	1.0738	0.170*	
H18C	0.4326	0.2322	0.9444	0.170*	
C19	0.3276 (4)	0.0840 (3)	0.5120 (4)	0.0867 (13)	
H19A	0.3532	−0.0069	0.5001	0.130*	
H19B	0.2833	0.0871	0.4281	0.130*	
H19C	0.2449	0.1017	0.5568	0.130*	

### Atomic displacement parameters (Å^2^)

**Table:**

	*U*^11^	*U*^22^	*U*^33^	*U*^12^	*U*^13^	*U*^23^
Br1	0.0726 (3)	0.0841 (3)	0.1278 (4)	0.0397 (2)	0.0242 (2)	0.0606 (3)
Br2	0.0993 (3)	0.0474 (2)	0.0780 (3)	0.02663 (18)	0.0233 (2)	0.03427 (17)
O1	0.0935 (16)	0.0611 (13)	0.0655 (14)	0.0282 (12)	0.0445 (13)	0.0362 (12)
O2	0.0505 (11)	0.0347 (10)	0.0600 (13)	0.0049 (9)	0.0086 (10)	0.0121 (9)
C1	0.0479 (16)	0.0516 (17)	0.069 (2)	0.0128 (14)	0.0002 (16)	0.0302 (16)
C2	0.064 (2)	0.0506 (18)	0.069 (2)	0.0021 (16)	−0.0034 (17)	0.0378 (17)
C3	0.070 (2)	0.0525 (17)	0.0519 (18)	0.0023 (16)	0.0104 (16)	0.0307 (15)
C4	0.0597 (17)	0.0444 (15)	0.0418 (16)	0.0028 (14)	0.0080 (14)	0.0199 (13)
C5	0.0469 (15)	0.0413 (14)	0.0388 (15)	0.0037 (12)	0.0019 (12)	0.0195 (12)
C6	0.0423 (15)	0.0500 (16)	0.0544 (18)	0.0049 (13)	0.0030 (13)	0.0261 (14)
C7	0.0532 (18)	0.074 (2)	0.082 (2)	0.0226 (16)	0.0233 (17)	0.0453 (19)
C8	0.0534 (17)	0.071 (2)	0.069 (2)	0.0182 (16)	0.0219 (16)	0.0454 (18)
C9	0.0450 (15)	0.0450 (15)	0.0470 (16)	0.0048 (12)	0.0066 (13)	0.0264 (13)
C10	0.0592 (18)	0.0503 (17)	0.060 (2)	−0.0016 (15)	−0.0029 (15)	0.0294 (15)
C11	0.067 (2)	0.0455 (16)	0.061 (2)	0.0013 (15)	0.0026 (17)	0.0274 (15)
C12	0.0552 (16)	0.0408 (14)	0.0401 (15)	0.0061 (13)	0.0114 (13)	0.0223 (12)
C13	0.0643 (18)	0.0431 (15)	0.0461 (16)	0.0183 (14)	0.0188 (15)	0.0273 (13)
C14	0.0542 (17)	0.0606 (19)	0.0566 (18)	0.0230 (16)	0.0177 (15)	0.0341 (16)
C15	0.0428 (15)	0.0515 (17)	0.0507 (17)	0.0052 (13)	0.0079 (13)	0.0219 (14)
C16	0.0488 (15)	0.0396 (14)	0.0392 (15)	0.0096 (12)	0.0152 (13)	0.0171 (12)
C17	0.0459 (14)	0.0409 (14)	0.0343 (14)	0.0087 (12)	0.0114 (12)	0.0195 (12)
C18	0.155 (4)	0.104 (3)	0.152 (5)	0.057 (3)	0.111 (4)	0.080 (3)
C19	0.060 (2)	0.0456 (19)	0.131 (4)	0.0056 (17)	0.026 (2)	0.015 (2)

### Geometric parameters (Å, °)

**Table:**

Br1—C1	1.907 (3)	C9—C17	1.515 (4)
Br2—C13	1.903 (3)	C9—C10	1.551 (4)
O1—C4	1.364 (4)	C10—C11	1.539 (4)
O1—C18	1.417 (4)	C10—H10A	0.9700
O2—C16	1.367 (3)	C10—H10B	0.9700
O2—C19	1.408 (4)	C11—C12	1.502 (4)
C1—C2	1.368 (5)	C11—H11A	0.9700
C1—C6	1.389 (4)	C11—H11B	0.9700
C2—C3	1.381 (5)	C12—C17	1.384 (4)
C2—H2A	0.9300	C12—C13	1.386 (4)
C3—C4	1.386 (4)	C13—C14	1.367 (4)
C3—H3A	0.9300	C14—C15	1.387 (4)
C4—C5	1.392 (4)	C14—H14A	0.9300
C5—C6	1.390 (4)	C15—C16	1.390 (4)
C5—C9	1.517 (3)	C15—H15A	0.9300
C6—C7	1.493 (4)	C16—C17	1.386 (4)
C7—C8	1.538 (4)	C18—H18A	0.9600
C7—H7A	0.9700	C18—H18B	0.9600
C7—H7B	0.9700	C18—H18C	0.9600
C8—C9	1.554 (4)	C19—H19A	0.9600
C8—H8A	0.9700	C19—H19B	0.9600
C8—H8B	0.9700	C19—H19C	0.9600
			
C4—O1—C18	117.9 (3)	C9—C10—H10A	110.5
C16—O2—C19	118.4 (2)	C11—C10—H10B	110.5
C2—C1—C6	120.7 (3)	C9—C10—H10B	110.5
C2—C1—Br1	119.1 (2)	H10A—C10—H10B	108.7
C6—C1—Br1	120.1 (3)	C12—C11—C10	102.8 (2)
C1—C2—C3	120.1 (3)	C12—C11—H11A	111.2
C1—C2—H2A	120.0	C10—C11—H11A	111.2
C3—C2—H2A	120.0	C12—C11—H11B	111.2
C2—C3—C4	120.4 (3)	C10—C11—H11B	111.2
C2—C3—H3A	119.8	H11A—C11—H11B	109.1
C4—C3—H3A	119.8	C17—C12—C13	119.5 (3)
O1—C4—C3	124.6 (3)	C17—C12—C11	110.9 (2)
O1—C4—C5	116.1 (2)	C13—C12—C11	129.6 (3)
C3—C4—C5	119.3 (3)	C14—C13—C12	120.5 (2)
C6—C5—C4	120.2 (2)	C14—C13—Br2	119.9 (2)
C6—C5—C9	111.3 (2)	C12—C13—Br2	119.6 (2)
C4—C5—C9	128.4 (3)	C13—C14—C15	120.3 (3)
C1—C6—C5	119.2 (3)	C13—C14—H14A	119.9
C1—C6—C7	129.8 (3)	C15—C14—H14A	119.9
C5—C6—C7	111.0 (2)	C14—C15—C16	120.0 (3)
C6—C7—C8	103.2 (2)	C14—C15—H15A	120.0
C6—C7—H7A	111.1	C16—C15—H15A	120.0
C8—C7—H7A	111.1	O2—C16—C17	116.2 (2)
C6—C7—H7B	111.1	O2—C16—C15	124.6 (2)
C8—C7—H7B	111.1	C17—C16—C15	119.2 (2)
H7A—C7—H7B	109.1	C12—C17—C16	120.6 (2)
C7—C8—C9	106.4 (2)	C12—C17—C9	111.2 (2)
C7—C8—H8A	110.5	C16—C17—C9	128.2 (2)
C9—C8—H8A	110.5	O1—C18—H18A	109.5
C7—C8—H8B	110.5	O1—C18—H18B	109.5
C9—C8—H8B	110.5	H18A—C18—H18B	109.5
H8A—C8—H8B	108.6	O1—C18—H18C	109.5
C17—C9—C5	118.2 (2)	H18A—C18—H18C	109.5
C17—C9—C10	101.5 (2)	H18B—C18—H18C	109.5
C5—C9—C10	111.8 (2)	O2—C19—H19A	109.5
C17—C9—C8	111.6 (2)	O2—C19—H19B	109.5
C5—C9—C8	101.4 (2)	H19A—C19—H19B	109.5
C10—C9—C8	112.7 (2)	O2—C19—H19C	109.5
C11—C10—C9	106.1 (2)	H19A—C19—H19C	109.5
C11—C10—H10A	110.5	H19B—C19—H19C	109.5
			
C6—C1—C2—C3	−1.1 (5)	C17—C9—C10—C11	−26.4 (3)
Br1—C1—C2—C3	−178.9 (2)	C5—C9—C10—C11	−153.3 (3)
C1—C2—C3—C4	0.4 (5)	C8—C9—C10—C11	93.2 (3)
C18—O1—C4—C3	−3.1 (5)	C9—C10—C11—C12	25.7 (3)
C18—O1—C4—C5	176.9 (4)	C10—C11—C12—C17	−15.2 (3)
C2—C3—C4—O1	−179.0 (3)	C10—C11—C12—C13	164.8 (3)
C2—C3—C4—C5	1.0 (4)	C17—C12—C13—C14	0.0 (4)
O1—C4—C5—C6	178.4 (3)	C11—C12—C13—C14	180.0 (3)
C3—C4—C5—C6	−1.5 (4)	C17—C12—C13—Br2	178.7 (2)
O1—C4—C5—C9	0.8 (4)	C11—C12—C13—Br2	−1.3 (4)
C3—C4—C5—C9	−179.2 (3)	C12—C13—C14—C15	−0.5 (4)
C2—C1—C6—C5	0.5 (5)	Br2—C13—C14—C15	−179.2 (2)
Br1—C1—C6—C5	178.3 (2)	C13—C14—C15—C16	0.3 (4)
C2—C1—C6—C7	−179.3 (3)	C19—O2—C16—C17	176.6 (3)
Br1—C1—C6—C7	−1.5 (5)	C19—O2—C16—C15	−3.3 (4)
C4—C5—C6—C1	0.8 (4)	C14—C15—C16—O2	−179.7 (3)
C9—C5—C6—C1	178.8 (2)	C14—C15—C16—C17	0.5 (4)
C4—C5—C6—C7	−179.4 (3)	C13—C12—C17—C16	0.8 (4)
C9—C5—C6—C7	−1.4 (3)	C11—C12—C17—C16	−179.2 (3)
C1—C6—C7—C8	165.1 (3)	C13—C12—C17—C9	178.2 (2)
C5—C6—C7—C8	−14.7 (4)	C11—C12—C17—C9	−1.8 (3)
C6—C7—C8—C9	24.5 (3)	O2—C16—C17—C12	179.1 (2)
C6—C5—C9—C17	138.9 (3)	C15—C16—C17—C12	−1.0 (4)
C4—C5—C9—C17	−43.4 (4)	O2—C16—C17—C9	2.1 (4)
C6—C5—C9—C10	−103.8 (3)	C15—C16—C17—C9	−178.0 (3)
C4—C5—C9—C10	74.0 (4)	C5—C9—C17—C12	140.4 (3)
C6—C5—C9—C8	16.5 (3)	C10—C9—C17—C12	17.7 (3)
C4—C5—C9—C8	−165.7 (3)	C8—C9—C17—C12	−102.6 (3)
C7—C8—C9—C17	−151.6 (3)	C5—C9—C17—C16	−42.4 (4)
C7—C8—C9—C5	−24.9 (3)	C10—C9—C17—C16	−165.1 (3)
C7—C8—C9—C10	94.8 (3)	C8—C9—C17—C16	74.6 (3)

### Hydrogen-bond geometry (Å, °)

**Table:**

*D*—H···*A*	*D*—H	H···*A*	*D*···*A*	*D*—H···*A*
C18^i^—H18A^i^···O1	0.96	2.56	3.416 (6)	149
C19^ii^—H19A^ii^···O2	0.96	2.52	3.365 (2)	147

Symmetry codes: (i) −*x*+1, −*y*+1, −*z*+2; (ii) −*x*+1, −*y*, −*z*+1.
